# Proteomic Analysis Revealed Metabolic Inhibition and Elongation Factor Tu Deamidation by *p*-Coumaric Acid in *Cronobacter sakazakii*

**DOI:** 10.3389/fmicb.2022.888103

**Published:** 2022-05-09

**Authors:** Ping Lu, Xuemeng Ji, Juan Xue, Yinping Dong, Xi Chen

**Affiliations:** ^1^Tianjin Key Laboratory of Ophthalmology and Visual Science, Tianjin Eye Institute, Tianjin Eye Hospital, Tianjin, China; ^2^Nankai University Affiliated Eye Hospital, Nankai University, Tianjin, China; ^3^Clinical College of Ophthalmology, Tianjin Medical University, Tianjin, China; ^4^Tianjin Key Laboratory of Food Science and Health, School of Medicine, Nankai University, Tianjin, China; ^5^Institute of Infection and Immunity, Taihe Hospital, Hubei University of Medicine, Shiyan, China; ^6^Institute of Radiation Medicine, Chinese Academy of Medical Science, Peking Union Medical College, Tianjin, China; ^7^State Key Laboratory of Medicinal Chemical Biology, Nankai University, Tianjin, China

**Keywords:** *p*-coumaric acid, *Cronobacter sakazakii*, proteome, deamidation, elongation factor Tu

## Abstract

Screening drugs and compounds to fight against *Cronobacter sakazakii* (*C. sakazakii*), one of the most common pathogens that can cause fatal necrotizing enterocolitis, septicema and meningitis, is still needed. We found that *p*-coumaric acid (pCA) has an inhibitory effect on *C. sakazakii in vitro* and *in vivo*. Proteomic changes of *C. sakazakii* BAA-894 exposed to pCA were studied to reveal the antibacterial mechanisms involved. A total of 1,553 proteins were identified in *C. sakazakii* BAA-894 by label-free proteomics analysis. Fuzzy cluster analysis showed that 33 were up-regulated, and 110 were down-regulated with pCA treatment. Gene Ontology (GO) analysis concluded that pCA caused the change of metabolic state of bacteria and generally in the state of metabolic inhibition. KEGG Enrichment Analysis (KEGG) analysis showed that pCA inhibited energy metabolism and distorted the balance of amino acid metabolism. Posttranslational modification analysis showed that pCA affected the deamidation of three proteins, including Elongation factor Tu, one of the vital proteins in bacteria. Molecular docking suggested the hydrogen bond between the pCA carboxyl group and Elongation factor Tu Asn-64 might contribute to deamidation. Overall, we found that pCA interfered with cellular energy and amino acid metabolism and promoted elongation factor Tu deamidation, suggesting that pCA can be an effective natural substitute to control *C. sakazakii*.

## Introduction

*Cronobacter sakazakii* is a Gram-negative bacterium formerly known as “*Enterobacter sakazakii*”. *Cronobacter sakazakii* can cause severe meningitis, sepsis, and necrotizing enterocolitis (Ji et al., 2021b). This microbe is ubiquitous in the environments and is often isolated from water, soil, kitchens, vegetables, and a variety of food samples, including powdered infant formula (Mohammed et al., 2015; Li et al., 2016; Henry and Fouladkhah, 2019). *Cronobacter sakazakii* infections can occur in all age groups, and most, though usually not serious, infections occur in adults (Kadlicekova et al., 2018; Holý et al., 2019; Ohira et al., 2021). Nevertheless, immunocompromised patients, as well as premature, low-birth-weight babies were considered at higher risk (Stoll et al., 2004). Many cases of *C. sakazakii* infection have been reported worldwide in neonatal intensive care units. Mortality rates range from 40 to 80%, and meningitis survivors often suffer serious complications, including hydrocephalus, quadriplegia, and neural retardation (Nazarowec-White and Farber, 1997; Corti et al., 2007; Lachowska et al., 2021).

*Cronobacter sakazakii* is becoming a thorny problem, not least because of the broad distribution of the pathogen. Nearly half of the cases of *C. sakazakii* infection have been traced to contaminated infant formula, where the existence of *C. sakazakii* is now strictly limited (Iversen and Forsythe, 2003). However, given the wide distribution of *C. sakazakii* and its strong adaptability to the environment, it is challenging to control *C. sakazakii* and this may lead to occasional recalls of powdered infant formula (Var et al., 2021). Another reason is the emergence of drug-resistance of *C. sakazakii*, isolated in many countries, including environmental and clinical isolates, to vancomycin, penicillin, oxacillin, lincosamides, etc., details are as follows: in India, *C. sakazakii* isolated from newborns was resistant to cephalosporin, fluoroquinolones, and aminoglycosides (Kakatkar et al., 2017). Resistant *C. sakazakii* strains resistant to penicillin, tetracycline, ciprofloxacin, and nalidixic acid were isolated from kitchen samples in Tennessee, United States (Kilonzo-Nthenge et al., 2012), and multiple resistant strains were reported in China, causing a severe meningitis case in one neonate (Zeng et al., 2018).

The emergence of multidrug-resistant strains has prompted the researchers to turn their attention to traditional medicine to find alternatives. Volatile oils from leaves and flowers were reported to have antibacterial activity against *C. sakazakii* (Sharma and Prakash, 2013). The water-soluble muscadine seed extracts can inactivate *C. sakazakii* (Kim et al., 2009). Trans-cinnamaldehyde (TC), an ingredient in cinnamon, can control *C. sakazakii* (Amalaradjou and Venkitanarayanan, 2011). TC also inhibits biofilm synthesis and thus can be used to prevent *C. sakazakii* biofilming on infant formula feeding equipment.

A growing body of studies strongly suggest that phenolic compounds have antimicrobial activity (Basgedik et al., 2014; Ceylan and Alıc, 2015; Călinoiu and Vodnar, 2020). Our previous studies have shown that *p*-coumaric acid (pCA), a phenolic acid of the hydroxycinnamic acid family, can inhibit growth and promote plasmid elimination of *C. sakazakii* (Ji et al., 2021a). However, the mechanism of pCA inhibiting the growth of *C. sakazakii* was lacking.

Our study used proteomics to characterize protein profile changes in *C. sakazakii* exposed to coumaric acid in an attempt to find the antibacterial mechanism of pCA. At the same time, posttranslational modifications were analyzed to find a possible direct interaction between pCA and proteins. At last, molecular docking was performed to study the potential molecular interactions.

## Materials and Methods

### Bacterial Strains and Growth Conditions

International standard strain *C. sakazakii* BAA-894 (ATCC, United States) was used in this study. Strain was stored in Luria-Bertani (LB) media (Oxoid, United Kingdom) containing 15% glycerol (Biosharp, China) at −80°C. To initiate all experiments, one loop of strain was innoculated in LB and cultured overnight. And then bacterium was diluted in LB media containing different concentrations of pCA to 10^6^ CFU/mL; the growth or inactivation of bacteria was monitored by serial dilution of bacteria and plating on LB agar. The plates were incubated at 37°C for 24–48 h, and then colony forming unit (CFU) was numbered.

### *In vivo* Rat Virulence Assay

Bacterial cells were washed and resuspended in phosphate buffered saline (PBS) (Vazyme, China). The bacterial suspension (5 × 10^9^ CFU) were administered by gavage to 3-day-old female Sprague Dawley rats (6/group). Where appropriate, pCA was administered at a dose of 1 mg/g weight 2 h after bacteria challenging, while an equal volume of pCA was administered without bacterial inoculation as the negative infection control and bacterial inoculation but without pCA treatment as positive infection control. Rats were maintained in their home cages in the animal house at 24 ± 1°C and 55 ± 5% humidity, with a 12-h light-dark cycle (light on at 8:00 and off at 20:00) To analyze the colonization of bacteria in blood and organs, the rats were sacrificed after 24 h of injection; organs were homogenized in ice-cold PBS and serially diluted. Bacterial load was determined by plating the diluents on LB agar.

### Protein Extraction

The bacterial suspension from *in vitro* experiment was centrifuged at 4°C for 2 min at 12,000g and washed twice with PBS. A protease inhibitor cocktail (Beyong, China) was added, and the bacteria were lysed by ultrasound (200 W, working 3 s and pausing 3 s) for 10 min. The protein extractions were centrifuged to obtain the supernatant (21,000g, 15 min at 4°C). Clean up the protein using a microporous filter (0.22 μm), and the extracted protein was stored at –80°C for subsequent MS analysis.

### Protein Digestion

The protein samples for LC-MS/MS analysis were prepared according to the protocols described previously with minor modifications (Xue et al., 2022). Specifically, the protein sample (50 μg, qualified by optical density at 260 nm) was denatured by adding one-fourth 8M urea (Aladdin, China). Add 50 mM ammonium bicarbonate (Aladdin, China) to make the total volume reach 100 μL. Incubate the mixture at 37°C for 30 min. Subsequently, the protein sample was reducted by reacting with 1 μL 200 mM Dithiothreitol (DTT) (Aladdin, China) and incubated for 30 min at 60°C. Cooldown to room temperature for 5 min. The protein was alkylated by adding 1 μL 500 mM iodoacetamide (Aladdin, China). Keep shaking for 30 min without light. Then quench iodoacetamide by adding DTT (1 μL, 200 mM) and vortex for 10 min. The samples were subsequently digested by trypsin (Thermo, United States) (4 μL, 0.25 μg/μL) at 37°C overnight. Terminate digestion by adding 10% trifluoroacetic acid (Aladdin, China) to the final concentration of 0.4%. At last, the samples were desalted with a C18 SPE column (Millipore, United States) and dried under vacuum. The vacuum-dried samples were submitted for MS analysis.

### LC-MS/MS Analysis

The digested peptide samples were analyzed using a Q Exactive Plus mass spectrometer and an EASY nano Liquid chromatography (EASY nLC 1200, Thermo Scientific) with an EASY nanoelectrospray interface according to the methods described before (Xue et al., 2022). The nano liquid chromatography system was equipped with a Thermo Scientific Acclaim Pepmap nano-trap column (C18, 5 μm, 100 Å, 100 μm × 2 cm) and a Thermo Scientific EASY-Spray column (Pepmap RSLC, C18, 2 μm, 100 Å, 50 μm × 15 cm). The nano liquid chromatography used solvent A (0.1% formic acid) and solvent B (80% CH_3_CN/0.1% formic acid), whose gradients were as follows: 0–8% B for 3 min, 8–28% B for 42 min, 28–38% B for 5 min, 38–100% B for 10 min. The mass spectra were searched against the UniProt database, and the MS raw data for each sample were searched using Maxquant (Version 2.0.3.1). Related parameters and instructions were as follows: samples with carbamidomethylation of cysteine set as a fixed modification. Oxidation (M) is set as the variable modifications. Searches were performed with trypsin cleavage specificity allowing two miscleavage events. The precursor mass tolerance was set to 10 parts-per-million (ppm) and a fragment mass tolerance of 0.02 Da. A maximum false discovery rate (FDR) of 1.0% was set for protein and peptide identifications. Protein identification was based on at least one unique peptide identification. Protein quantification was calculated as the median of unique peptides of the protein.

### Molecular Docking

The crystal structures of all the proteins used in the docking were predicted based on Alphafold2 (Jumper et al., 2021); the 3D structures of the small molecules were downloaded from the PubChem database (Kim et al., 2016), and the Chem3D V20 was used to minimize the energy in the MMFF94 field (Hajos, 2002). AutoDock Vina 1.1.2 was used to perform molecular docking (Gaillard, 2018). Before docking, PyMOL 2.5 was used to treat all receptor proteins, including removing water molecules, salt ions, and other small molecules (Skern, 2018). Then set up the docking box using the PyMOL plug and define the center of mass of the active site residue as the center of the docking box with the side length set to 22.5 angstroms. In addition, use ADFRsuite 1.0 to convert all processed small molecules and receptor proteins into the PDBQT format required for AutoDock Vina 1.1.2 docking. When docking, the global search verbosity is set to 20, leaving the rest of the parameters set by default. The output of the highest-scoring docking conformations is considered to be the binding conformations, and finally, PyMol is used for visual analysis of docking results.

### Statistical Analysis

All experiments were conducted in triplicate, as independent experiments. Gene Ontology (GO) and KEGG Enrichment Analysis (KEGG) analysis was performed on DAVID’s online website^1^ and enrichment scores were calculated automatically (Dennis et al., 2003). Fuzzy C-means (FCM) algorithm was conducted with R (Bezdek et al., 1984; Ihaka and Gentleman, 1996). Statistical analyses were performed with GraphPad Prism software or R as described in Experimental Procedures for individual analysis. For all, *p* < 0.05 was considered statistically significant.

## Results

### Bacteriostatic Effect of p-Coumaric Acid

**FIGURE 1:**
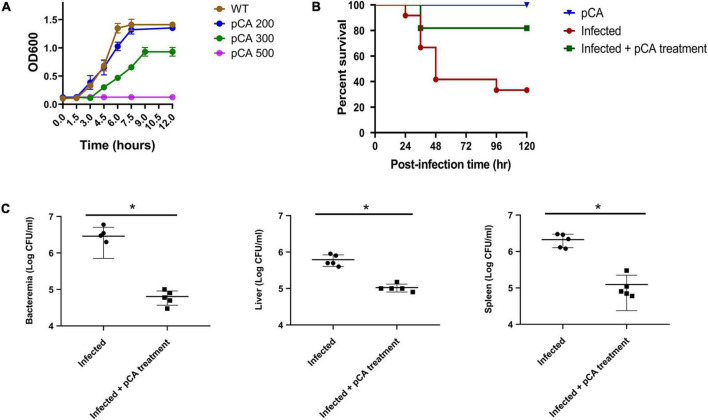
The effect of p-coumaric acid (pCA) on *Cronobacter sakazakii in vitro* and *in vivo*. **(A)** Growth curves of *C. sakazakii* under three different concentrations (mg/L) of pCA (*n* = 3). **(B)** Survival of infected rats (*n* = 12). In the pCA treatment group, the puppies received gavage treatment of pCA (1 mg/g weight) 2 h after bacterial infection, while an equal volume of saline was administered as the control. The rats received gavage treatment of pCA (1 mg/g weight) as uninfected control. **(C)** The bacterial load of *C. sakazakii* in the blood, kidney, and spleen of the rat 1 day after infection (*n* = 4). Student’s *t* test was used to test whether there is a difference between two independent sample. *p* < 0.05 was considered statistically significant. *Significant differences (T test, *p* < 0.05).

The effect of pCA on the growth of *C. sakazakii* is shown in Figure 1A. The bacteria in the control group began to grow exponentially after 20 mins and reached stationary phase at 6 h. *Cronobacter sakazakii* was inhibited at the concentration of 300 μg/mL pCA, and reached the stationary phase after 9 h. When the concentration of pCA reached 500 μg/mL, the growth of bacteria was inhibited entirely. These results indicated that pCA could inhibit the growth of *C. sakazakii in vitro*. We also studied the effect of pCA *in vivo* (Figure 1B). Rats were used as the infection model. In the pCA treatment group, the rats received gavage treatment of pCA (1 mg/g weight) 2 h after bacterial infection, while an equal volume of saline was administered as the control. The results showed that pCA could significantly improve the survival rate of infected rats. Moreover, pCA treatment significantly reduced the bacterial density of *C. sakazakii* in the blood, liver, and spleen of the rat sacrificed 1 day after infection (Figure 1C). Taken together, these results suggest that pCA can effectively inhibit bacterial growth *in vivo* and *in vitro*.

### Proteomic Analysis of *Cronobacter sakazakii* Exposure to p-Coumaric Acid

**FIGURE 2:**
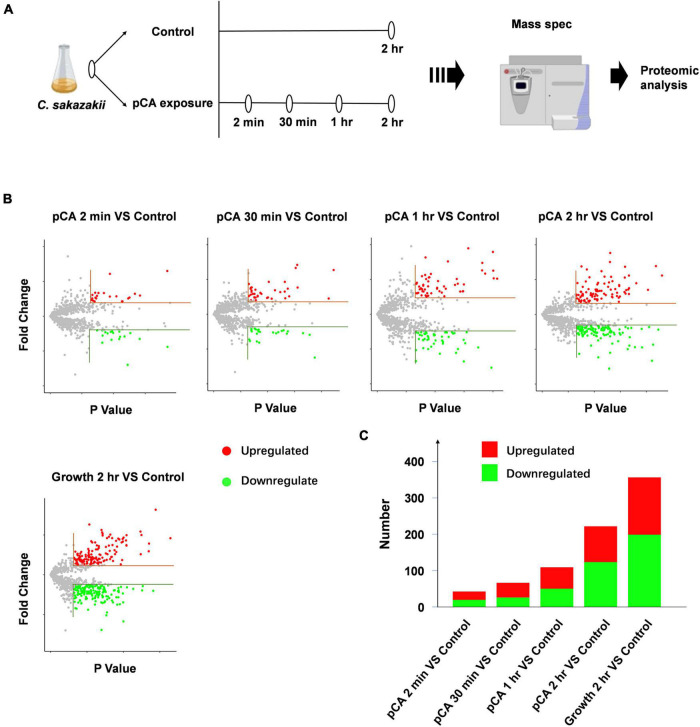
Mass spec and proteomic analysis of *C. sakazakii* exposure to pCA. **(A)** Schematic of Mass spec experiment. *Cronobacter sakazakii* at 2 mins, 30 mins, 1 h, and 2 h after pCA exposure were sampled for Mass spec experiment. *Cronobacter sakazakii* not exposed to pCA at the initial stage and after 2 h of normal growth was set as controls (*n* = 3). **(B)** Volcano plot depicting proteins that significantly increase (red) or decrease (green) abundance with exposure time (Supplementary Table 1). Proteins not affected are shown in gray. The horizontal line indicates an absolute fold change (Abs > 2) and vertical dashed lines a significance cutoff of *q* < 0.05. **(C)** The number of proteins that significantly increase (red) or decrease (green) abundance in panel **(B)** (Supplementary Table 1).

**FIGURE 3:**
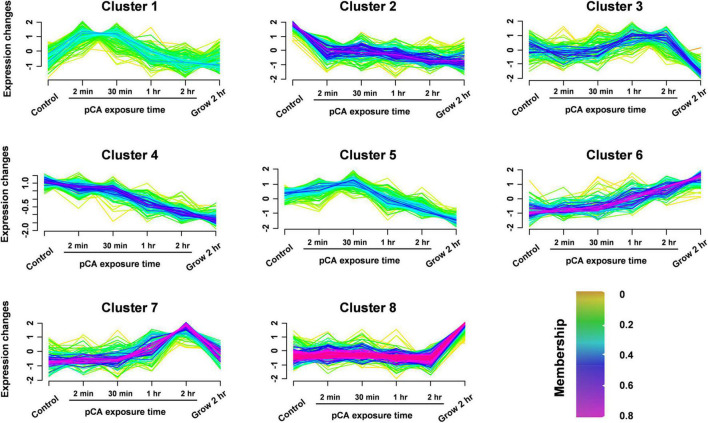
Clustering the protein expression profiles at different stages using Fuzzy C-means (FCM) algorithm. The expression profiles were clustered into eight different regulatory patterns, indicating different expression dynamics. See also Supplementary Table 2.

Using label-free relative quantitative mass spectrometry, we systematically monitored the protein expression profiles of *C. sakazakii* at 2 mins, 30 mins, 1 h, and 2 h after pCA exposure (Figure 2A). *C. sakazakii* not exposed to pCA at the initial stage and after 2 h of normal growth were set as controls. We obtained 15,621 unique peptides that matched 1,553 proteins. Among them, 905 proteins met the quantitative requirements (Figure 3A). *T*-test (*p* < 0.05) was used to determine the up-and down-regulated peptides according to the criterion that the folding change of differential expressed proteins was more than two times (Figure 2B and Supplementary Table 1). Compared with the control group, 2 mins of pCA exposure could significantly change 39 protein abundances, and abundance changes increased over prolonged exposure time to pCA (Figure 2C). After 2 h of pCA treatment, there were 90 up-regulated proteins and 113 down-regulated proteins. Surprisingly, however, the bacterial proteome showed even more changes after 2 h in standard growth control. It is suggested that prolonged time and drug treatment can cause changes in the proteome.

To distinguish the pCA-induced proteome changes from time-induced proteome changes, fuzzy C-means (FCM) algorithm was used to cluster the protein expression profiles at different time stages (Figure 3 and Supplementary Table 2). In general, we observed eight different time-pattern clusters representing different regulatory proteins, indicating different expression dynamics. Clusters 2 and 4 represent down-regulated proteins, clusters 6 and 7 represent up-regulated proteins, clusters 1, 3, and 5 represent proteins showing bimodal expression patterns, and cluster 8 characterizes proteins which expression does not change much. When time interference was excluded by superimposing the protein expression 2 h after normal growth, some proteins were continuously upregulated or downregulated, clustered in clusters 7 and 8, respectively. The up- and down-regulated proteins accounted for 11 and 17% of the total 905 proteins. Among the core-altered proteins (membership > 0.58), 40% were downregulated, 3.3 times as many as the upregulated. Overall, we found that pCA caused protein expression changes and more downregulated proteins than upregulated proteins, consistent with the observed inhibition of bacterial growth exposed to pCA.

### Fuzzy Cluster and Gene Ontology Analysis

**FIGURE 4:**
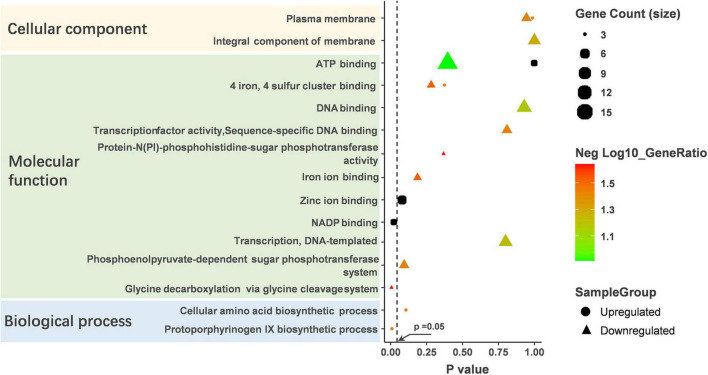
GO analysis on the up-regulated and down-regulated proteins. GO analysis was performed on DAVID’s online website (https://david.ncifcrf.gov) and enrichment scores were calculated automatically. *p* < 0.05 was considered statistically significant.

GO analysis revealed that the altered proteins were enriched in the molecular function (Figure 4 and Supplementary Table 3). The NADP binding and glycine decarboxylation via glycine cleavage system were significantly enriched among molecular function clusters. The upregulated protein was significantly enriched in NADP binding. NADP-binding proteins are associated with the metabolic status of bacteria, so we hypothesize that pCA causes changes in the metabolic status of bacteria. In addition, pCA-induced up-regulated proteins were enriched in the biological process of the Protoporphyrinogen IX biosynthesis, and Protoporphyrinogen IX was reported to play an essential role in the biosynthesis of heme, suggesting that the bacteria were in an iron-deficient state. It is well documented that polyphenols can chelate iron ions, and perhaps pCA can competitively chelate environmental iron ions, making it difficult for bacteria to obtain iron. Additionally, pCA-induced down-regulated proteins were enriched in seven biological-function-related clusters and one cellular component-related cluster. However, pCA-up-regulated proteins were enriched in only two clusters. Both up-regulated and down-regulated proteins were found in three clusters: the plasma membrane, ATP binding, four iron, and four sulfur cluster binding. Still, the number of down-regulated proteins was more than up-regulated in the same cluster. Collectively, GO analysis infers that pCA leads to alterations in the bacterial metabolic state and an overall metabolically repressed state.

### KEGG Enrichment Analysis Pathway Analysis

**FIGURE 5:**
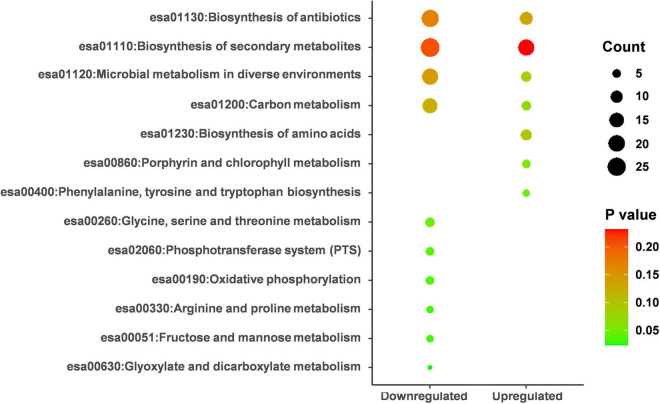
KEGG pathway analysis on the up-regulated and down-regulated proteins. KEGG analysis was performed on DAVID’s online website (https://david.ncifcrf.gov) and enrichment scores were calculated automatically. *p* < 0.05 was considered statistically significant.

By performing KEGG pathway enrichment analysis, the down-regulated proteins were significantly enriched in seven pathways (*p* < 0.05), and the up-regulated proteins were enriched in only one pathway (Figure 5 and Supplementary Table 3). pCA distorted L-phenylalanine, tyrosine, and tryptophan biosynthesis. Of the seven pathways down-regulated, “metabolism” was the most common, including several subclasses: “glyoxylic and dicarboxylic acid metabolism,” “fructose and mannose metabolism,” “arginine and proline metabolism,” “glycine, serine and threonine metabolism.” In addition, some important categories were also significantly enriched (e.g., “phosphotransferase system,” “oxidative phosphorylation”). These results illustrated that pCA might inhibit cellular energy metabolism while the balance of amino acid metabolism might be disturbed.

### Deamidation of Proteins by p-Coumaric Acid

Three proteins were identified that were affected by pCA to increase deamidation significantly. These include: A7MKI5, which has 64 amino acid L-asparagine deamidation, A7MGU5: 43 amino acid L-asparagine deamidation, A7MKM7:379 amino acid L-asparagine deamidation. Notably, all three proteins have important effects on the physiological functions of bacteria. A7MKI5 is a crucial protein of bacteria, termed Elongation factor Tu (EF-TU), a G protein with the intrinsic ability to hydrolyze GTP into GDP, contributes to the overall translation fidelity, and plays a vital role in the elongation process of extension of protein biosynthesis. And A7MGU5 is an ancillary SecYEG translocon subunit, which is thought to act as a molecular chaperone, helps maintain the typical conformation of proteins in bacteria. A7MKM7 is a Peptidase M3 domain-containing protein with zinc ion metal-binding ability and metallopeptidase activity. In summary, we found that the effect of pCA on posttranslational modifications mainly affected the deamidation of bacteria, including three critical proteins.

### Molecular Docking Study

**FIGURE 6:**
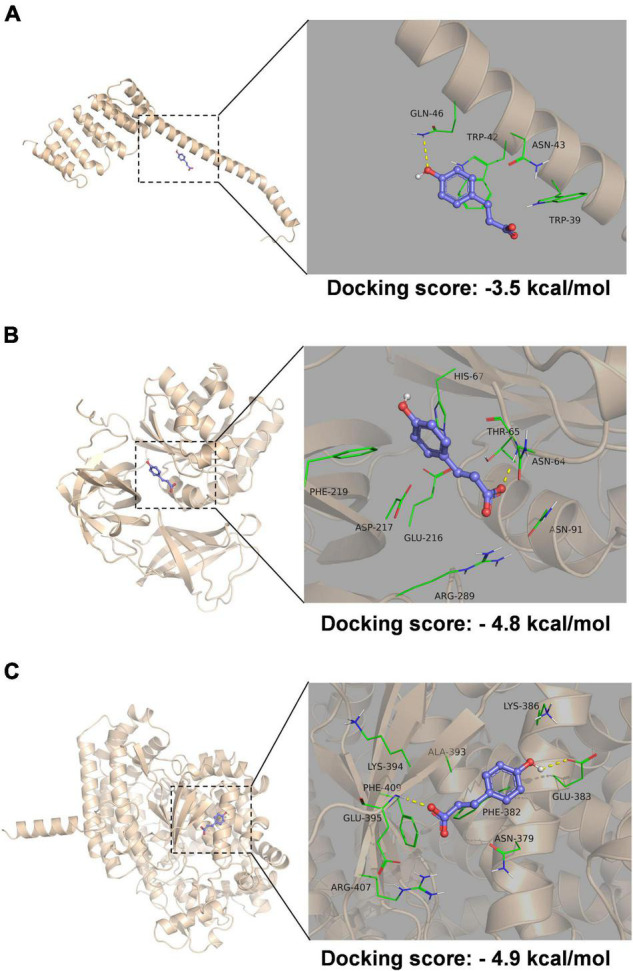
Molecular docking of protein and pCA complex. Molecular docking of A7MGU5/pCA complex **(A)**, A7MKI5/pCA complex **(B)**, and A7MKM7/pCA complex **(C)**. The picture on the left is the overall view, and the picture on the right is the partial view. In the picture, the blue stick is the small molecule pCA, the wheat color cartoon is a protein, the green represents the amino acids around the binding site, and the yellow dotted line represents hydrogen bonding.

Molecular docking was performed to help understand the structure-based correlation between the deamidation of proteins and the structure of pCA (Figure 6). The structures of the three proteins affected by deamidation were predicted using alpha fold, and then molecular docking results were obtained using autodock Vina. We obtained the binding affinities of individual combinations. A negative binding affinity indicates the presence of binding. The binding energy of A7MKM7/pCA was −4.9 kcal/mol, followed by A7MKI5/pCA with a binding energy of −4.8 kcal/mol and A7MGU5/pCA complex, the binding energy is −3.5 kcal/mol. As shown in Figure 6A, in the A7MGU5/pCA complex, pCA was in contact with Gln-46, Trp-42, Asn-43, and Trp-39 on a long helix, where phenolic hydroxyl forms a hydrogen bond with Gln-46 The aromatic ring forms a pipi-stack action with the Trp directly below. In the A7MKI5/pCA complex (Figure 6B), pCA was contact with Phe-219, Glu-216, Asp-217, Arg-289, Asn-91, Asn-64, and Thr-65 on the A7MKI5 protein, in which the carboxyl group forms a hydrogen bond with the amide bond on the backbone of asn-64, which is consistent with the deamidation site analyzed by the proteomic data. It also includes hydrophobic interaction with Phe-219 and Glu-216. In the A7MKM7/pCA complex (Figure 6C), pCA came into contact with Glu-383, Lys-386, Lys-394, Glu-395, Arg-407, Phe-409, Ala-393, and Phe-382 on the A7MKM7 protein, the phenolic hydroxyl group formed a hydrogen bond with Glu-383, and the carboxyl group formed a hydrogen bond with amide bond on the main chain of GLU-395. Hydrophobic interaction formed with Phe-409, Ala-393, and Phe-382.

## Discussion

Previous studies have shown that pCA can kill bacteria through a dual damage mechanism, increasing membrane permeability and binding to DNA phosphate anions (Lou et al., 2012). These mechanisms affect replication, transcription, and protein expression. Here we report the effect of pCA on the proteomic changes of *C. sakazakii*.

In this study, label-free protein mass spectrometry was used to investigate the proteomic changes caused by pCA. pCA caused most downregulation of core-altered proteins and a few proteins upregulated. This result is not surprising because pCA suppresses bacterial growth. The reduced proteins caused by pCA were significantly enriched in the glycine decarboxylation via glycine cleavage system, which widely exists in plants, animals, and microorganisms and is associated with metabolism and glycine tolerance. It has been found that glycine decarboxylase mutants can cause metabolic disorders in microalgae, glycine accumulation, and death, and in *Streptomyces griseus*, the deactivation of glycine lytic system will lead to sensitivity to glycine (Tezuka and Ohnishi, 2014). Thus, downregulation of the glycine decarboxylation via glycine cleavage system by pCA may have caused the derangement of glycine-related metabolism in bacteria. Interestingly, we found a protein, PgmA, which abundance increased with the time of pCA exposure (data not shown). Whether increased PgmA can resulted in negative impact on bacterial growth remains to be further studied. Considering many substances inhibit bacteria by disrupting cell morphology and increasing cell membrane permeability (Chitemerere and Mukanganyama, 2014; Shi et al., 2016), the possible association of PgmA with cell morphology and cell membrane permeability needs to be taken into account.

In addition to the regulation of biological metabolic pathways, protein post-translational modifications by chemicals are also important but often overlooked. Protein posttranslational modification includes many kinds, such as acetylation, methylation, etc.; some of these modifications may even affect host-bacteria interactions, such as the recently reported CSPC acetylation modification that enhances the presence of Pseudomonas aeruginosa in the host (Li et al., 2021). We characterized the post-translational effects of pCA, including acetylation, phosphorylation, methylation, and deamidation. However, except for changes in deamidation, we did not identify any other type of significant modification changes. Our study found that pCA induced deamidation of elongation factor Tu. These results suggest that the antibacterial action of pCA on *C. sakazakii* is multi-target (Zhang et al., 2021). But whether *C. sakazakii* is easy to develop resistance to pCA needs further study.

Our posttranscriptional modification analysis combined with molecular docking revealed that pCA has a targeted deamidation effect on the elongation factor Tu. The A7MKI5/*p*-Coumaric complex predictes the hydrogen bonding of the pCA carboxyl group to the amide bond on the ASN-64 backbone, which is consistent with the deamidation sites analyzed by the proteomic data, which further confirms the potential interaction between Elongation factor Tu and pCA. Considering the critical role of elongation factor Tu for microorganisms, the deamidation of the factor by pCA may cause the growth inhibition of *C. sakazakii*. This is the first report investigated the deamidation effect of bacteriostatic drugs on proteins in organisms, and also the first report on the deamidation effect of bacteriostatic drugs on elongation factor Tu. The importance of the elongation factor Tu for microorganisms has long been recognized, and research has been underway to develop antibiotics that inhibit its effects. As far in 1974, the inhibitory effect of kirromycin on the bacterial elongation factor Tu has been found (Wolf et al., 1974). Over the past few decades, a great deal of biological information has been accumulated about Elongation factor Tu inhibitors. Recently, the Elongation factor-Tu has developed into an attractive antibacterial target for rational drug discovery (Harvey et al., 2019). However, to sum up, the currently reported drugs, including rationally designed drugs, are primarily targeted into the pocket of the elongation factor Tu (Parmeggiani and Nissen, 2006; Parmeggiani et al., 2006; Kumar and Garg, 2022). Deamidation seems not to be the active modification of bacteria but the degradation process of proteins. For example, active protein drugs will undergo spontaneous deamidation during storage, and deamidation may affect the normal function of drugs, but the deamidation in organisms is still lacking research (Liu, 1992; Krause and Sahin, 2019). Our study suggests that the asparagine deamidation of the elongation factor Tu may also be a target for antimicrobial agents.

In conclusion, our study demonstrates on the one hand that pCA interferes with cellular energy and amino acid metabolism, thus inhibiting bacterial growth. On the other hand, we found that pCA promoted L-asparagine deamidation of the bacterial elongation factor Tu, which hints at the great potential of deamidation of elongation factor Tu as an antimicrobial target.

## Data Availability Statement

The mass spectrometry proteomics data have been deposited to the ProteomeXchange Consortium (http://proteomecentral.proteomexchange.org) via the iProX partner repository (Ma et al., 2019) with the dataset identifier PXD032021.

## Ethics Statement

The animal study was reviewed and approved by Institutional Animal Care and Use Committee of Nankai University.

## Author Contributions

PL conceived and designed the research. JX and YD contributed new reagents or analytical tools. XC and JX conducted the experiments. XC and XJ take charge of analyzing the data, research and wrote the manuscript. All authors contributed to the editing of the manuscript and approved the submitted version.

## Conflict of Interest

The authors declare that the research was conducted in the absence of any commercial or financial relationships that could be construed as a potential conflict of interest.

## Publisher’s Note

All claims expressed in this article are solely those of the authors and do not necessarily represent those of their affiliated organizations, or those of the publisher, the editors and the reviewers. Any product that may be evaluated in this article, or claim that may be made by its manufacturer, is not guaranteed or endorsed by the publisher.

## Footnotes

1
https://david.ncifcrf.gov

## References

[B1] Amalaradjou M. A. R. Venkitanarayanan K. (2011). Effect of trans-cinnamaldehyde on inhibition and inactivation of Cronobacter sakazakii biofilm on abiotic surfaces. *J. Food Protect.* 74 200–208. 10.4315/0362-028X.JFP-10-296 21333138

[B2] Basgedik B. Ugur A. Sarac N. (2014). Antimicrobial, antioxidant, antimutagenic activities, and phenolic compounds of Iris germanica. *Industr. Crops Prod.* 61 526–530. 10.1093/jpp/rgab008 33772287

[B3] Bezdek J. C. Ehrlich R. Full W. (1984). FCM: the fuzzy c-means clustering algorithm. *Comput. Geosci.* 10, 191–203. 10.1016/0098-3004(84)90020-7

[B4] Călinoiu L. F. Vodnar D. C. (2020). Thermal processing for the release of phenolic compounds from wheat and oat bran. *Biomolecules* 10:21. 10.3390/biom10010021 31877857 PMC7023188

[B5] Ceylan O. Alıc H. (2015). Antibiofilm, Antioxidant, Antimutagenic Activities and Phenolic Compounds of Allium orientale BOISS. *Braz. Arch. Biol. Technol.* 58 935–943. 10.1590/s1516-89132015060309

[B6] Chitemerere T. A. Mukanganyama S. (2014). Evaluation of cell membrane integrity as a potential antimicrobial target for plant products. *BMC Complementary Altern. Med.* 14:278. 10.1186/1472-6882-14-278 PMC4124163 25078023

[B7] Corti G. Panunzi I. Losco M. Buzzi R. (2007). Postsurgical osteomyelitis caused by *Enterobacter sakazakii* in a healthy young man. *J. Chemother.* 19 94–96. 10.1179/joc.2007.19.1.94 17309858

[B8] DennisG.ShermanB. T.HosackD. A.YangJ.GaoW.LaneH. C. (2003). DAVID: database for annotation, visualization, and integrated discovery. *Genom. Biol.* 4:P3.12734009

[B9] Gaillard T. (2018). Evaluation of AutoDock and AutoDock Vina on the CASF-2013 benchmark. *J. Chem. Inform. Modeling* 58 1697–1706. 10.1021/acs.jcim.8b00312 29989806

[B10] Hajos Z. (2002). *Proline Catalyzed Asymmetric Cyclization. Theory of the Reaction Mechanism. Science Direct Working Paper(S1574-0331): 04.* New York: SSRN.

[B11] Harvey K. L. Jarocki V. M. I. Charles G. Djordjevic S. P. (2019). The diverse functional roles of elongation factor Tu (EF-Tu) in microbial pathogenesis. *Front. Microbiol.* 10:2351. 10.3389/fmicb.2019.02351 31708880 PMC6822514

[B12] Henry M. Fouladkhah A. (2019). Outbreak history, biofilm formation, and preventive measures for control of Cronobacter sakazakii in infant formula and infant care settings. *Microorganisms* 7:77. 10.3390/microorganisms7030077 30870985 PMC6463179

[B13] Holý O. Cruz-Córdova A. Xicohtencatl-Cortes J. Hochel I. Parra-Flores J. Petrželová J. (2019). Occurrence of virulence factors in Cronobacter sakazakii and Cronobacter malonaticus originated from clinical samples. *Microb. Pathog.* 127 250–256. 10.1016/j.micpath.2018.12.011 30550840

[B14] Ihaka R. Gentleman R. (1996). R: a language for data analysis and graphics. *J. Comput. Graph. Stat.* 5, 299–314. 10.2307/1390807

[B15] Iversen C. Forsythe S. (2003). Risk profile of *Enterobacter sakazakii*, an emergent pathogen associated with infant milk formula. *Trends Food Sci. Technol.* 14 443–454. 10.1016/s0924-2244(03)00155-9

[B16] Ji X. Lu P. Xue J. Zhao N. Zhang Y. Dong L. (2021b). The lipoprotein NlpD in Cronobacter sakazakii responds to acid stress and regulates macrophage resistance and virulence by maintaining membrane integrity: running Title: identification and characterization of a novel factor involved in acid tolerance and virulence in Cronobacter sakazakii. *Virulence* 12 415–429. 10.1080/21505594.2020.1870336 33459158 PMC7834084

[B17] Ji X. Lu P. Hu Y. Xue J. Wu J. Zhang B. (2021a). Function Characterization of Endogenous Plasmids in Cronobacter sakazakii and Identification of p-Coumaric Acid as Plasmid-Curing Agent. *Front. Microbiol.* 12:687243. 10.3389/fmicb.2021.687243 34248908 PMC8267800

[B18] Jumper J. Evans R. Pritzel A. Green T. Figurnov M. Ronneberger O. (2021). Highly accurate protein structure prediction with AlphaFold. *Nature* 596 583–589. 34265844 10.1038/s41586-021-03819-2 PMC8371605

[B19] Kadlicekova V. Kajsik M. Soltys K. Szemes T. Slobodnikova L. Janosikova L. (2018). Characterisation of Cronobacter strains isolated from hospitalised adult patients. *Antonie van Leeuwenhoek* 111 1073–1085. 10.1007/s10482-017-1008-2 29270766

[B20] Kakatkar A. S. Gautam R. K. Godambe P. L. Shashidhar R. (2017). Culture dependent and independent studies on emerging food-borne pathogens Cronobacter sakazakii, *Klebsiella pneumoniae* and Enterococcus faecalis in Indian food. *Int. Food Res. J.* 24 2645–2651.

[B21] Kilonzo-Nthenge A. Rotich E. Godwin S. Nahashon S. Chen F. (2012). Prevalence and antimicrobial resistance of Cronobacter sakazakii isolated from domestic kitchens in middle Tennessee, United States. *J. Food Protect.* 75 1512–1517. 10.4315/0362-028X.JFP-11-442 22856579

[B22] Kim S. Thiessen P. A. Bolton E. E. Chen J. Fu G. Gindulyte A. (2016). PubChem substance and compound databases. *Nucleic Acids Res.* 44 D1202–D1213. 26400175 10.1093/nar/gkv951 PMC4702940

[B23] Kim T. J. Silva J. L. Weng W. L. Chen W. W. Corbitt M. Jung Y. S. (2009). Inactivation of *Enterobacter sakazakii* by water-soluble muscadine seed extracts. *Int. J. Food Microbiol.* 129 295–299. 10.1016/j.ijfoodmicro.2008.12.014 19167124

[B24] Krause M. E. Sahin E. (2019). Chemical and physical instabilities in manufacturing and storage of therapeutic proteins. *Curr. Opin. Biotechnol.* 60 159–167. 10.1016/j.copbio.2019.01.014 30861476

[B25] Kumar N. Garg P. (2022). Probing the Molecular Basis of Cofactor Affinity and Conformational Dynamics of Mycobacterium tuberculosis Elongation Factor Tu: an Integrated Approach Employing Steered Molecular Dynamics and Umbrella Sampling Simulations. *J. Phys. Chem. B* 126 1447–1461. 10.1021/acs.jpcb.1c09438 35167282

[B26] Lachowska M. Izdebski R. Urbanowicz P. Żabicka D. Królak-Olejnik B. (2021). Infection of Cronobacter sakazakii ST1 Producing SHV-12 in a Premature Infant Born from Triplet Pregnancy. *Microorganisms* 9:1878. 10.3390/microorganisms9091878 34576773 PMC8469300

[B27] Li S. Weng Y. Li X. Yue Z. Chai Z. Zhang X. (2021). Acetylation of the CspA family protein CspC controls the type III secretion system through translational regulation of exsA in *Pseudomonas aeruginosa*. *Nucleic Acids Res.* 49 6756–6770. 10.1093/nar/gkab506 34139014 PMC8266623

[B28] Li Z. Ge W. Li K. Gan J. Zhang Y. Zhang Q. (2016). Prevalence and characterization of Cronobacter sakazakii in retail milk-based infant and baby foods in Shaanxi, China. *Foodborne Pathog. Dis.* 13 221–227. 10.1089/fpd.2015.2074 26886843

[B29] Liu D. T.-Y. (1992). Deamidation: a source of microheterogeneity in pharmaceutical proteins. *Trends Biotechnol.* 10 364–369. 10.1016/0167-7799(92)90269-2 1368876

[B30] Lou Z. Wang H. Rao S. Sun J. Ma C. Li J. (2012). p-Coumaric acid kills bacteria through dual damage mechanisms. *Food Control* 25 550–554. 10.1016/j.foodcont.2011.11.022

[B31] Ma J. Chen T. Wu S. Yang C. Bai M. Shu K. (2019). iProX: an integrated proteome resource. *Nucleic Acids Res.* 47 D1211–D1217. 10.1093/nar/gky869 30252093 PMC6323926

[B32] Mohammed M. A. Sallam K. I. Tamura T. (2015). Prevalence, identification and molecular characterization of Cronobacter sakazakii isolated from retail meat products. *Food Control* 53 206–211. 10.1016/j.foodcont.2015.01.010

[B33] Nazarowec-White M. Farber J. M. (1997). *Enterobacter sakazakii*: a review. *Int. J. Food Microbiol.* 34 103–113. 9039558 10.1016/s0168-1605(96)01172-5

[B34] Ohira S. Ikeda E. Kamijo K. Nagai T. Tsunemi K. Uchiyama N. (2021). Pyosalpinx due to Cronobacter sakazakii in an elderly woman. *BMC Womens Health* 21:136. 10.1186/s12905-021-01283-8 33794866 PMC8017632

[B35] Parmeggiani A. I Krab M. Watanabe T. Nielsen R. C. Dahlberg C. Nyborg J. (2006). Enacyloxin IIa pinpoints a binding pocket of elongation factor Tu for development of novel antibiotics. *J. Biol. Chem.* 281 2893–2900. 10.1074/jbc.M505951200 16257965

[B36] Parmeggiani A. Nissen P. (2006). Elongation factor Tu-targeted antibiotics: four different structures, two mechanisms of action. *FEBS Lett.* 580 4576–4581. 10.1016/j.febslet.2006.07.039 16876786

[B37] Sharma G. Prakash A. (2013). Susceptibility of Cronobacter sakazakii to plant products, antibiotics, and to lactic acid bacteria. *Int. J. Nutr. Pharmacol. Neurol. Dis.* 3:263. 10.4103/2231-0738.114847

[B38] Shi C. Sun Y. I. Zheng Z. Zhang X. Song K. Jia Z. (2016). Antimicrobial activity of syringic acid against Cronobacter sakazakii and its effect on cell membrane. *Food Chem.* 197 100–106. 10.1016/j.foodchem.2015.10.100 26616929

[B39] Skern T. (2018). *An Archive and a Tool: pdb and pymol. Exploring Protein Structure: Principles and Practice.* Berlin: Springer, 7–28.

[B40] Stoll B. J. Hansen N. Fanaroff A. A. Lemons J. A. (2004). *Enterobacter sakazakii* is a rare cause of neonatal septicemia or meningitis in VLBW infants. *J. Pediatr.* 144 821–823. 10.1016/j.jpeds.2004.02.045 15192634

[B41] Tezuka T. Ohnishi Y. (2014). Two glycine riboswitches activate the glycine cleavage system essential for glycine detoxification in Streptomyces griseus. *J. Bacteriol.* 196 1369–1376. 10.1128/JB.01480-13 24443533 PMC3993345

[B42] Var I. Özçakmak S. Tekin A. Yılmaz S. Heshmati B. Uçkun O. (2021). Evaluation of Food Safety of Commercial Baby Foods according to Legal Regulations. *Eur. J. Agric. Food Sci.* 3 72–80. 10.24018/ejfood.2021.3.5.373

[B43] Wolf H. Chinali G. Parmeggiani A. (1974). Kirromycin, an inhibitor of protein biosynthesis that acts on elongation factor Tu. *Proc. Natl. Acad. Sci. U.S.A.* 71 4910–4914. 10.1073/pnas.71.12.4910 4373734 PMC434009

[B44] Xue J. Huang Y. Zhang H. Hu J. Pan X. Peng T. (2022). Arginine GlcNAcylation and Activity Regulation of PhoP by a Type III Secretion System Effector in *Salmonella*. *Front. Microbiol.* 12:825743. 10.3389/fmicb.2021.825743 35126337 PMC8811161

[B45] Zeng H. Lei T. He W. Zhang J. Liang B. Li C. (2018). Novel multidrug-resistant Cronobacter sakazakii causing meningitis in neonate, China, 2015. *Emerg. Infect. Dis.* 24:2121. 10.3201/eid2411.180718 30334728 PMC6199977

[B46] Zhang Y. Liu Y. Tang Y. Zhang D. He H. Wu J. (2021). Antimicrobial α-defensins as multi-target inhibitors against amyloid formation and microbial infection. *Chem. Sci.* 12 9124–9139. 10.1039/d1sc01133b 34276942 PMC8261786

